# Draft Genome Sequences of Enterotoxigenic *Bacillus cereus* Strains Obtained from Powdered Infant Formula

**DOI:** 10.1128/genomeA.01644-16

**Published:** 2017-02-23

**Authors:** Laurenda Carter, Hannah R. Chase, Hyerim Choi, SoYoung Jun, JiHyeon Park, Seungeun Jeong, MiJeong Kim, KyuYoung Han, ChaeYoon Lee, HyeJin Jeong, Samantha Finkelstein, Flavia Negrete, Hediye N. Cinar, Ben D. Tall, Gopal R. Gopinath

**Affiliations:** Center for Food Safety and Applied Nutrition, U.S. Food and Drug Administration, Laurel, Maryland, USA

## Abstract

We introduce the draft genome sequences of five enterotoxigenic *Bacillus cereus* strains: Bc 12, Bc 67, Bc 111, Bc 112, and Bc 113, which were obtained from powdered infant formula. The genome sizes of the strains ranged from 5.5 to 5.8 Mb, and the G+C contents were ~35.2%.

## GENOME ANNOUNCEMENT

*Bacillus cereus*, a spore-forming Gram-positive bacterium, is widely distributed in the environment and often occurs in foods, including low-water-content or ready-to-eat foods (1–5). It can produce multiple heat-labile diarrhea-causing enterotoxins. *B. cereus* has been implicated in the frequent contamination of dried milk-based products, a topic recently related by Reyes et al. (6), who reported high levels of *B. cereus* contamination in infant formula served to children in Chile. Although other food-related *B. cereus* genome sequences have been reported (7), very little genomic information on *B. cereus* strains isolated from powdered infant formula is available.

DNA was isolated from *B. cereus* strains Bc 12, Bc 67, Bc 111, Bc 112, and Bc 113 using a Mo Bio UltraClean microbial DNA isolation kit (Mo Bio Laboratories, Inc., Carlsbad, CA), according to the manufacturer’s instructions. Whole-genome sequencing (WGS) was performed using the MiSeq platform (Illumina, San Diego, CA, USA), and Illumina’s NextSeq XT library kit. Trimmed Fastq data sets were *de novo* assembled with CLC Genomics Workbench version 7.0 (CLC bio, Aarhus, Denmark). Information on the WGS assemblies is shown in Table 1; the assemblies were found to have between 81 and 262 contigs (≥500 bp long), 5,450,436 to 5,803,604 bases, ~35.2% G+C content, and between 5,628 and 6,078 coding sequences (CDS). WGS assemblies were annotated using the RAST annotation server (8).

**TABLE 1:** Genomic information on the enterotoxigenic *B. cereus* strains isolated from powdered infant formula

BioSample ID	Sample name	Genome size (bases)	No. of contigs	G+C content (%)	No. of CDSs	ST^a^	Accession no.
MOD1_Bc12	Bc 12	5,542,633	262	35.2	5,698	UD	MIFB00000000
MOD1_Bc67	Bc 67	5,803,604	91	35.2	6,078	205, CC205	MIFF00000000
MOD1_Bc111	Bc 111	5,520,811	115	35.2	5,686	32	MIFD00000000
MOD1_Bc112	Bc 112	5,504,580	96	35.2	5,628	127	MIFC00000000
MOD1_Bc113	Bc 113	5,450,436	81	35.3	5,682	205, CC205	MIFE00000000

a Sequence type (ST) was determined by uploading genome assemblies to http://mlstoslo.uio.no/. CC, clonal complex; UD, undetermined. The ST for the UD strain could not be determined because only five of the seven alleles matched an allele in the online MLST scheme and could possibly be either ST38 or ST1135.

Comparative genomic analysis showed that these strains possessed as many as 36 different alleles encoding efflux pumps (EP), including EP alleles for the major facilitator superfamily (MFS), resistance-nodulation-division (RND), and multidrug and toxic compound extrusion (MATE) EP families. Such EPs are involved in the transport of multiple antimicrobials, such as macrolides, heavy metals, and acriflavin, as well as the transport of heme, hemin, and formate. A global transcriptional regulator gene, *tet*(R), was found coupled to the RND multidrug efflux transporters. Not every EP family was found in each strain. For example, strain Bc 12 was the only strain that possessed an MFS EP, and strains Bc 67, Bc 111, and Bc 112, and Bc 111, Bc 112, and Bc 113 possessed a heme EP system and an RND efflux transport system, respectively. Strain Bc 12 possessed two alleles of a MATE EP, whereas strains Bc 67, Bc 112, and Bc 113 each possessed a single copy, and strain Bc 111 did not possess this gene at all. Other attributes found among the strains include genes encoding stress response proteins, such as heat shock proteins, stress response proteins 17 M and 26, a universal stress protein; *perR* of the FUR family; the transcriptional regulator *pspC*; several polysaccharide transferases and deacetylases; drug resistance genes for fosmidomycin, tetracycline, vancomycin (*vanW*), and daunorubicin; and *terD*, *emrE,* and *sugE*, which are involved in tellurium, ethidium bromide, and quaternary ammonium resistance, respectively. Interestingly, a gene encoding oxetanocin, a novel nucleoside, was found in all of the strains. This nucleoside was first described in *Bacillus megaterium* (9).

The data presented here increase the number of publically available *B. cereus* genomes, including, for the first time to our knowledge, genome sequences of isolates obtained from powdered infant formula. Further comparative genomic studies are warranted to ascertain their phylogenetic relatedness.

### Accession number(s).

The accession numbers for these *B. cereus* genome sequences are listed in Table 1 and were deposited at the National Center for Biotechnology Information under BioProject PRJNA326742 (*B. cereus* GenomeTrakr Project FDA-CFSAN).
